# Additive value of 3D-echo in prediction of immediate outcome after percutaneous balloon mitral valvuloplasty

**DOI:** 10.1186/s43044-019-0019-x

**Published:** 2019-09-18

**Authors:** Hazem M. A. Farrag, Amr M. Setouhi, Mustafa O. El-Mokadem, Mustafa A. El-Swasany, Khalid S. Mahmoud, Hesham B. Mahmoud, Alaa M. Ibrahim

**Affiliations:** 1 Cardiology Department, Faculty of Medicine, Minia University, Minya, 61111 Egypt; 2 Cardiology Department, Faculty of Medicine, Beni-Suef University, Beni Suef, Egypt; 3 Cardiology Department, Faculty of Medicine, El-Azhar University, Cairo, Egypt

**Keywords:** Mitral stenosis, Percutaneous balloon mitral valvuloplasty, Three-dimensional echocardiography, Mitral valve score, Multi-track balloon, Inoue balloon

## Abstract

**Background:**

Results of percutaneous balloon mitral valvuloplasty (BMV) are basically dependent on suitable patient selection. Currently used two-dimensional (2D) echocardiography (2DE) scores have many limitations. Three-dimensional (3D) echocardiography (3DE)-based scores were developed for better patient selection and outcome prediction. We aimed to compare between 3D-Anwar and 2D-Wilkins scores in mitral assessment for BMV, and investigate the additive value of 3DE in prediction of immediate post-procedural outcome. Fifty patients with rheumatic mitral stenosis and candidates for BMV were included. Patients were subjected to 2D- and real-time 3D-transthoracic echocardiography (TTE) before and immediately after BMV for assessing MV area (MVA), 2D-Wilkins and 3D-Anwar score, commissural splitting, and mitral regurgitation (MR). Transesophageal echocardiography (TEE) was also undertaken immediately before and intra-procedural. Percutaneous BMV was performed by either multi-track or Inoue balloon technique.

**Results:**

The 2DE underestimated post-procedural MVA than 3DE (*p* = 0.008). Patients with post-procedural suboptimal MVA or significant MR had higher 3D-Anwar score compared to 2D-Wilkins score (*p* = 0.008 and *p* = 0.03 respectively). The 3D-Anwar score showed a negative correlation with post-procedural MVA (*r* = − 0.48, *p* = 0.001). Receiver operating characteristic (ROC) curve analysis for both scores revealed superior prediction of suboptimal results by 3D-Anwar score (*p* < 0.0001). The 3DE showed better post-procedural posterior-commissural splitting than 2DE (*p* = 0.004). Results of both multi-track and Inoue balloon were comparable except for favorable posterior-commissural splitting by multi-track balloon (*p* = 0.04).

**Conclusion:**

The 3DE gave valuable additive data before BMV that may predict immediate post-procedural outcome and suboptimal results.

**Electronic supplementary material:**

The online version of this article (10.1186/s43044-019-0019-x) contains supplementary material, which is available to authorized users.

## Background

Mitral stenosis (MS) is a common valvular affection by the rheumatic process, for which percutaneous balloon mitral valvuloplasty (BMV) was firstly introduced by Inoue for treatment of selected patients, as a safe invasive procedure with equivalent or even better results as surgical commissurotomy [1, 2]. Nevertheless, safety and success of BMV techniques are mainly dependent on the selection of suitable patients. Patient ages, functional class, mitral valve (MV) morphology, pre-procedural MV area (MVA), and the size of the balloon used; all are considered as predictors of procedural outcome [3, 4]. Current scores that target patient selection before BMV—particularly the two-dimensional (2D) Wilkins score—may have many limitations including inability to assess commissural involvement or differentiate nodular fibrosis from calcification, ignoring the distribution of pathologic abnormalities and contribution of each variable and underestimating subvalvular disease [5]. Therefore, a new three-dimensional (3D) echocardiography (3DE) scoring system has been proposed by Anwar et al. for better guiding of MV intervention [6]. In the current study, we compared 3D-Anwar and 2D-Wilkins scores in assessment of MV before percutaneous BMV, and investigated the additive value of real-time 3DE in the prediction of immediate outcome of the procedure.

## Methods

The current study included 50 patients with rheumatic MS scheduled for percutaneous BMV, at the period from January 2017 to July 2018 Additional file 1.

### Inclusion criteria

Inclusion criteria are as follows: (1) significant MS [MVA < 1.5 cm^2^], (2) favorable MV score [Wilkins score < 11/16], and (3) isolated MS or associated with no more than mild mitral regurgitation (MR) or other valve lesions not requiring surgical correction.

### Exclusion criteria

The following criteria were basically excluded (1) MVA > 1.5 cm^2^, (2) associated moderate or severe MR or other valve lesions requiring surgical correction, (3) presence of left atrial (LA) and/or LA appendage thrombus, (4) history of recent thromboembolic events within the last month, (5) associated significant coronary artery disease requiring surgical revascularization, and (6) acute rheumatic activity and/or infective endocarditis [7]. The study protocol was approved by the institutional Ethical Committee and was in agreement with the “World Medical Association Declaration of Helsinki.” After obtaining assigned consent from every participating patient, all patients were subjected to history taking and clinical examination, resting 12-leads electrocardiogram (ECG). Two-dimensional transthoracic echocardiography (2D-TTE) assessment was performed to all patients before and after BMV using IE 33 X-Matrix echo machines and X5-1 matrix array transducers (2007 Koninklijke Philips Electronics N.V.) with harmonic imaging and continuous ECG monitoring. Assessment of the MVA by pressure half-time (PHT) and planimetry, trans-mitral gradient, commissural splitting, MR regurgitant-jet area, and peak systolic pulmonary artery pressure (PSPAP) through tricuspid regurgitation peak velocity were undertaken. MV Wilkins score was estimated, that depended on assessment of four parameters (MV leaflets’ mobility, thickness and calcification, and subvalvular involvement), and giving each parameter a score of 0–4. By calculating the sum of those scores, a total score of 0–16/16 (normal MV = 0/16, mild MV involvement = 1–4/16, moderate MV involvement = 5–8/16, and severe MV involvement > 8/16) was obtained [8–12]. Two-dimensional transesophageal echocardiography (2D-TEE) using multiplane probe was performed to all patients immediately before BMV in order to assess the LA and its appendage for evident thrombi or spontaneous echo contrast, thickness of the inter-atrial septum, MV morphology and score (especially the subvalvular apparatus), and measuring MV annulus to determine balloon size. 2D-TEE was also undertaken intra-procedurally to confirm balloon position and assess incremental change in trans-mitral gradient and commissure mobility with balloon inflation, and look for any increase in MR [13]. Real-time 3D-TTE was undertaken for all patients within 24–48 h before and immediately after BMV using Philips IE 33 X-matrix echo machine and X5-1 matrix-array transducer (2007 Koninklijke Philips Electronics N.V.) with continuous ECG monitoring. Image acquisition was done according to Lang et al. [14] for assessment of MV and estimating the MVA [15]. Pre-procedural commissural affection and post-procedural splitting were assessed, where fusion of both commissures (anterolateral and posteromedial) indicated no splitting, width of splitting < 0.5 cm indicating partial splitting, and width of splitting > 0.5 cm indicating complete splitting. Each commissure was separately assessed and the total score was calculated (0 for no splitting, 0.5 for partial splitting, and 1 for complete splitting) [6]. MV score was estimated according to Anwar et al., where scoring was done for both MV leaflets and chordal affection. Each leaflet was divided into three scallops (anterolateral, middle, and posteromedial), and each scallop was scored separately for thickness (0–1), mobility (0–1), and calcification (0–2 except for the middle scallop where score was 0–1). Both anterior and posterior chordae were assessed at three levels: proximal (valve level), middle, and distal (papillary muscle level). At each level, chordae were scored for thickness (0–1) and chordal separation (0–2). The sum of such scoring of leaflets’ mobility (0–6/6), thickness (0–6/6), and calcification (0–10/10), together with chordal thickness (0–3/3) and separation (0–6/6) can give the total score of 0–31/31 (normal MV = 0/31, mild MV involvement = 1–8 /31, moderate MV involvement = 9–13/31, and severe MV involvement ≥ 14/31) [6].

Percutaneous BMV procedure was undertaken using either Multi-track™ system (Numed Inc., Canada) or Inoue Balloon™ (Toray industries Inc., Japan) through trans-septal antegrade approach. Estimation of the balloon size for valvuloplasty was done by estimating the echocardiographic balloon reference sizing (3DE inter-commissural distance measured in mid-diastole), with the height-based balloon reference sizing (0.1 × height in centimeter + 10) was used for those with inconvenient echocardiographic balloon reference sizing. The required balloon size could be equal, increase, or decrease by one or two sizes as the estimated reference size [9]. Optimal post-procedural success was defined by achieving MVA ≥ 1.5 cm^2^ and/or 50% increase than the pre-procedural MVA, with less than moderate MR [7]. Obtaining suboptimal post-procedural MVA (i.e., < 1.5 cm^2^) and/or moderate or severe post-procedural MR were considered as suboptimal post-procedural outcome.

### Statistical analysis

Statistical analysis was performed using the statistical package for the social sciences (SPSS) software [version 17 for Windows] (SPSS Inc., Chicago, Illinois, USA). Continuous variables were expressed as number and (%) or mean ± standard deviation (SD). Comparison between variables (pre-procedural and post-procedural two-dimensional echocardiography (2DE) and 3DE data, also between post-procedural 2DE and 3DE data) was done using Student’s *t* test, while comparison of post-procedural commissural splitting data (between 2DE and 3DE, also between Inoue balloon group and multi-track balloon group) was done using chi-square tests. Z score was used for comparing between Wilkins and Anwar scores. Correlation of Wilkins and Anwar scores with achieved post-procedural MVA was done using Spearman rho correlation coefficient. Roc curve analysis was undertaken for Wilkins and Anwar scores in prediction of post-procedural suboptimal results. Statistical significance was defined as a probability level of *p* < 0.05.

## Results

**Fig. 1:**
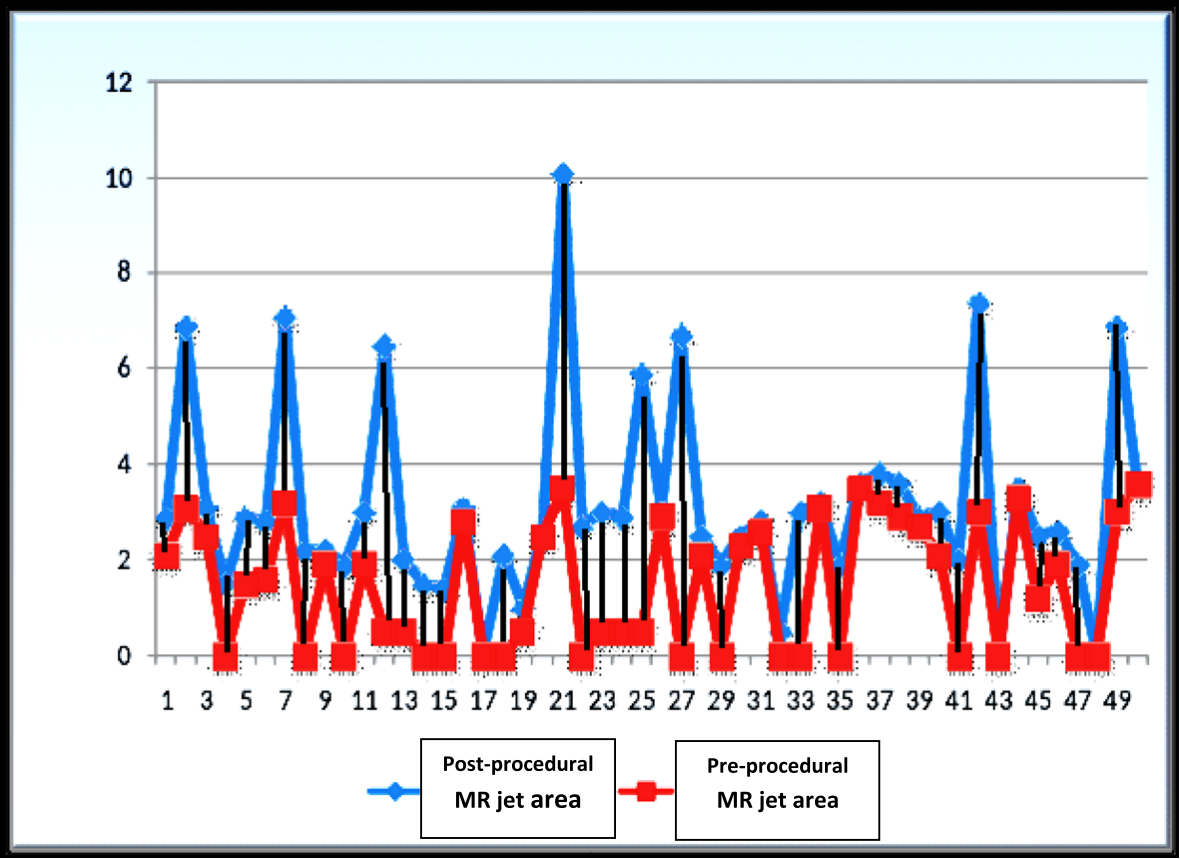
Change in mitral regurgitation jet area before and after percutaneous balloon mitral valvuloplasty in each case

The included 50 patients mean age was 29.5 ± 7.8 years (19–55 years old), and 64% of them were females. Thirty percent of patients had atrial fibrillation (AF), 66% were in New York Heart Association (NYHA) functional class III, while 18% were in class II and 16% were in class IV. No significant difference regarding pre-procedural MVA as estimated by PHT, 2D-, and 3D-planimetry (*p* = 0.244). Also, no statistical difference was observed regarding estimation of 2D-Wilkins score by TTE and immediate pre-procedural TEE (*p* = 0.176). Nevertheless, post-procedural MVA was underestimated by 2D-planimetry than by 3D-planimetry (*p* = 0.008). A significant increase was achieved in MVA after BMV (*p* < 0.0001), with significant reduction in mean and peak trans-mitral gradients and PSPAP (*p* < 0.0001 for all) (Table 1). Procedural success was obtained in 72% of cases, while 28% of cases had suboptimal post-procedural outcome (12% had suboptimal post-procedural MVA, and 16% had significant post-procedural MR). Fourteen percent of cases had no change between the pre- and post-procedural degree of MR, 70% developed new mild MR after BMV, while 14% and 2% had post-procedural moderate and severe MR respectively (Fig. 1).

**Table 1:** Change in MV gradients, PSPAP, and MV area before and after BMV

		Pre-procedural		Post-procedural		*P*
MV Mean gradient		19.8 ± 5.7		7.5 ± 3.8		< 0.0001
MV Max gradient		31.1 ± 8.5		16.1 ± 6.4		< 0.0001
PSPAP		57.6 ± 11.5		39.0 ± 8.3		< 0.0001
MV area	2D PHT	0.96 ± 0.16	*p* = 0.244	–	*p* = 0.008	< 0.0001
	2D planimetry	0.92 ± 0.15		1.70 ± 0.29		< 0.0001
	3D planimetry	0.97 ± 0.16		1.85 ± 0.36		< 0.0001

*MV* mitral valve, *PSPAP* peak systolic pulmonary artery pressure, *PHT* pressure half-time, *2D* 2-dimensional, *3D* 3-dimensional, *BMV* balloon mitral valvuloplasty

**Fig. 2:**
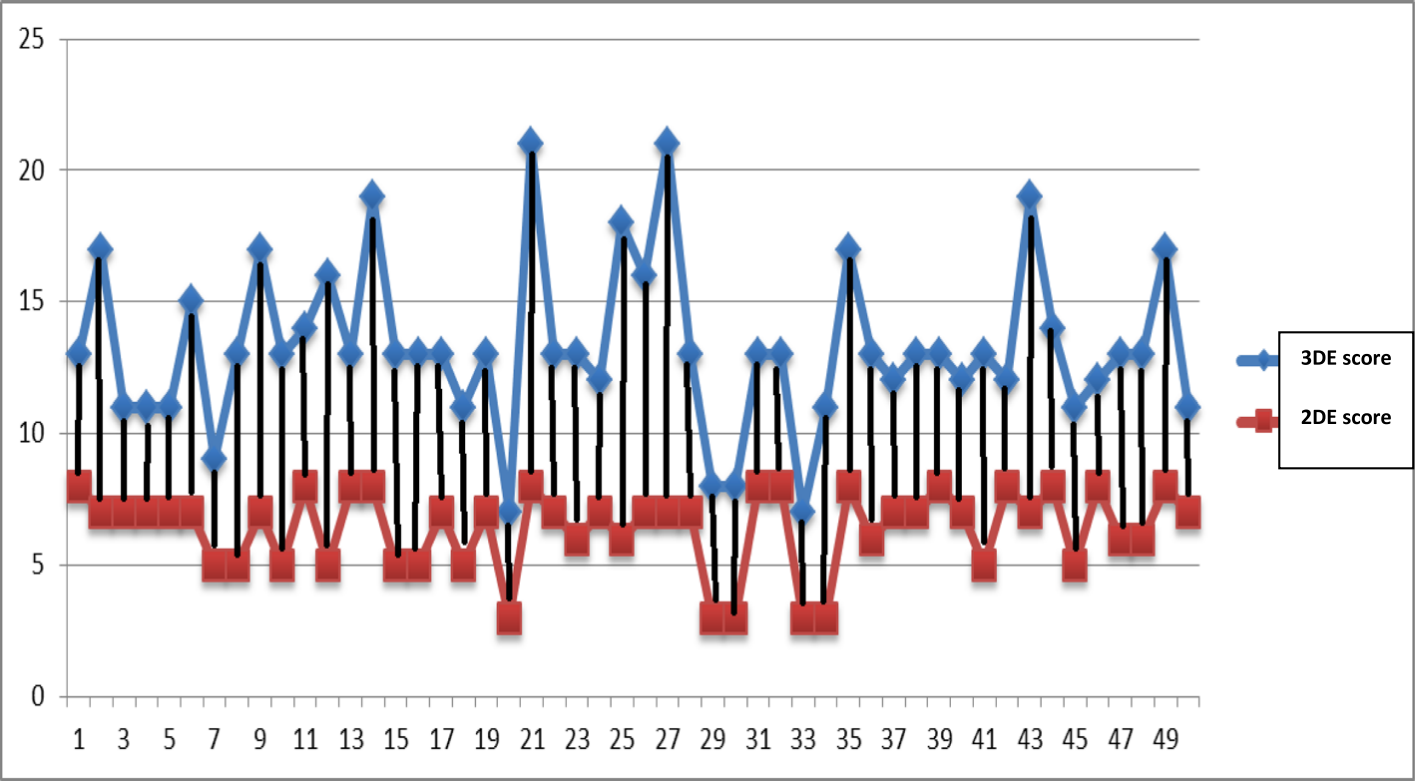
Pre-procedural 2D-Wilkins score and 3D-Anwar score in each case

**Fig. 3:**
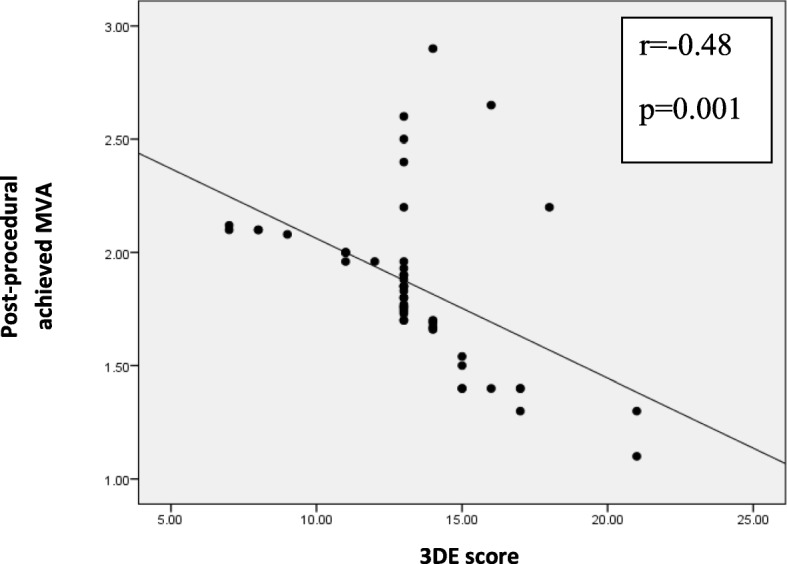
Correlation between 3D-Anwar score and achieved post-procedural mitral valve area

**Fig. 4:**
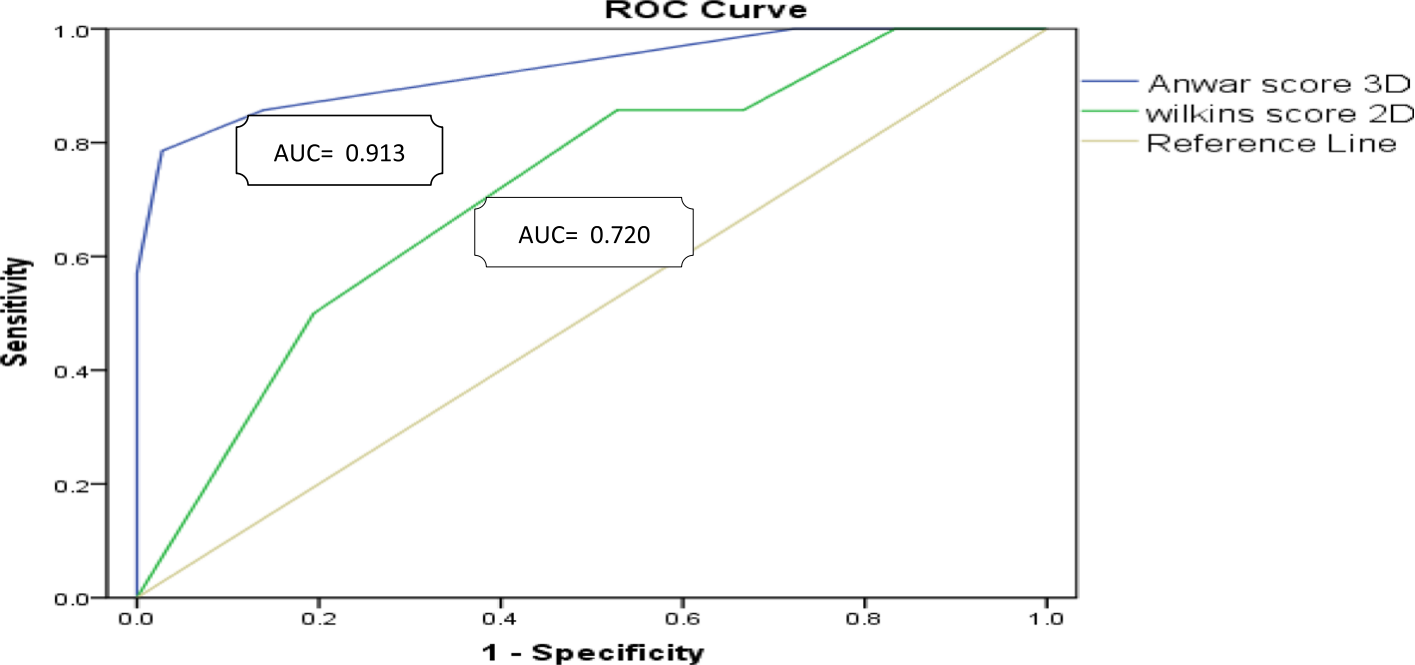
Roc curve analysis for 3D-Anwar score and 2D-Wilkins score in predicting the occurrence of suboptimal results after percutaneous balloon mitral valvuloplasty

Although no statistical difference were observed regarding assessment of total MV score and each of its components between 2D-Wilkins and 3D-Anwar scores in cases with optimal post-procedural results, but significantly higher total 3D-Anwar score in those with suboptimal results (*p* = 0.008 and *p* = 0.03 for suboptimal MVA and significant MR respectively), and that was also valid for each of its components (Table 2, Fig. 2). In the fourteen cases with suboptimal BMV outcome, eight cases had moderate total score and six cases had mild total score by 2D-Wilkins score, while 12 cases had severe total score and only two cases had moderate total score (with high scores for calcification and subvalvular affection) by 3D-Anwar score (*p* < 0.0001). Although the 3D-Anwar score showed weak negative correlation with the achieved post-procedural MVA (*r* = − 0.48, *p* = 0.001), the 2D-Wilkins score failed to show any correlation (*r* = − 0.14, *p* = 0.33) (Fig. 3). Moreover, receiver of characteristic (ROC) curve analysis revealed that the 3D-Anwar score was better than 2D-Wilkins score in predicting the occurrence of suboptimal results after BMV (area under curve (AUC) = 0.913, sensitivity = 76.9%, specificity = 94.6%, positive predictive value (PPV) = 83.3%, negative predictive value (NPV) = 92.1%, and *p* < 0.0001 vs. AUC = 0.720, sensitivity = 53.9%, specificity = 81.1%, PPV = 50%, NPV = 83.3%, and *p* = 0.01 respectively) (Fig. 4). Interestingly, the calculated mean post-procedural MVA for all cases (*n* = 50) vs. those with optimal achieved MVA only (i.e., with exclusion of cases of suboptimal achieved MVA) (*n* = 44) were 1.70 ± 0.29 cm^2^ vs. 2.2 ± 0.15 cm^2^ (as estimated by 2DE) and 1.85 ± 0.36 cm^2^ vs. 2.18 ± 0.21 cm^2^ (as estimated by 3DE).

**Table 2:** Comparison between 2D-Wilkins score and 3D=Anwar score in cases with post-procedural optimal results, suboptimal MV area, and significant MR

		2D	3D	*P*
Optimal post-procedural results (*n* = 36)	Mobility	2.02 ± 0.51	2.29 ± 1.11	0.9
	Thickness	1.88 ± 0.48	2.09 ± 0.74	0.5
	Subvalvular apparatus	1.82 ± 0.62	2.11 ± 1.12	0.7
	Calcification	0.79 ± 0.52	1.09 ± 1.37	0.1
	Total score	6.51 ± 0.87	7.58 ± 2.18	0.6
Suboptimal post-procedural MVA (*n* = 6)	Mobility	1.83 ± 0.41	4.16 ± 0.91	0.02
	Thickness	2.16 ± 0.41	4.66 ± 1.03	0.03
	Subvalvular apparatus	2.17 ± 0.41	7.01 ± 0.89	0.01
	Calcification	0.83 ± 0.75	3.33 ± 1.03	0.007
	Total score	6.99 ± 1.01	19.16 ± 3.86	0.008
Significant post-procedural MR (*n* = 8)	Mobility	1.75 ± 0.71	3.37 ± 0.91	0.014
	Thickness	1.88 ± 0.64	3.50 ± 0.53	0.001
	Subvalvular apparatus	1.75 ± 0.46	4.12 ± 0.64	0.01
	Calcification	1.25 ± 0.71	5.01 ± 0.75	0.01
	Total score	7.79 ± 1.12	16.0 ± 2.83	0.03

*2D* 2-dimensional, *3D* 3-dimensional, *MV* mitral valve, *MR* mitral regurgitation

*P* value for comparison between both scores was estimated by Z score

There was a significant improvement in anterior and posterior commissural splitting after BMV (*p* = 0.001), with better post-procedural posterior commissural splitting as assessed by 3DE than by 2DE (*p* = 0.004) (Table 3).

**Table 3:** Pre-procedural commissural affection and post-procedural commissural splitting as estimated by 2D and 3D echocardiography

		Pre-procedural Commissural affection			Post-procedural Commissural splitting		
		2D	3D	*p*	2D	3D	*p*
Anterior commissure	No	46 (92%)	47 (94%)	0.93	8 (16%)	4 (8%)	0.18
	Partial	4 (8%)	3 (6%)	0.6	28 (56%)	24 (48%)	0.4
	Complete	0	0	–	14(28%)	22 (44%)	0.9
Posterior commissure	No	47 (94%)	46 (92%)	0.93	12 (24%)	5 (10%)	0.07
	Partial	3 (6%)	4 (8%)	0.6	31 (62%)	25 (50%)	0.8
	Complete	0	0	–	7 (14.0%)	20 (40%)	0.004

*2D* 2-dimensional, *3D* 3-dimensional

Regarding type of balloon used, 34 cases (68%) had BMV using multi-track balloon, while 16 cases (32%) by Inoue balloon. In multi-track balloon group, 21, seven, and six cases had balloon size equal, − 1, and + 2 the estimated reference balloon sizing respectively, while in Inoue balloon group, 12, two, and two cases had balloon size equal, − 1, and + 2 the estimated reference balloon sizing respectively. Multi-track balloon showed better post-procedural posterior commissural splitting than Inoue balloon as estimated by 3DE (14.7%, 67.6%, 17.6% vs. 43.8%, 50%. 6.3% for no, partial, and complete splitting by multi-track vs. Inoue balloon respectively, *p* = 0.04), and no statistical difference was observed between both balloon techniques regarding achieved post-procedural MVA (*p* = 0.12) or MR grade (*p* = 0.24)**.** Interestingly, all cases with suboptimal MVA (*n* = 6) were done using + 2 the estimated reference balloon sizing, while all those with significant MR were done using equal, − 1, and − 2 the estimated reference balloon sizing (*n* = 2, 3, 3 respectively).

## Discussion

The ideal MV echocardiographic scoring system must consider the following:

- Qualitative and quantitative global and segmental evaluation of each component of the MV apparatus separately.
- Inclusion of all points that proved to predict and affect the outcome of BMV.
- Ease of application and interpretation within a reasonable time, with high reproducibility and reliability.
- Validity for both transthoracic and transesophageal approaches [16].

The currently used 2D-Wilkins score had basically depended on assessment of four parameters (MV leaflets’ mobility, thickness and calcification, and subvalvular involvement), and although its limitations that have been noted for a long time therefore many modifications were developed [17–19]; Wilkins scores ≤ 8/16 persisted as an independent predictor of acute success after BMV in a recent study conducted by Meneguz-Moreno et al. on a very long-term follow-up after percutaneous BMV [20]. The 3DE enables a comprehensive anatomic characterization of leaflet, commissural, and chordal involvement from any unconventional plane [15]. A novel 3D scoring system for MV was introduced by Anwar et al., which basically depended on scoring of both MV leaflets and chordal affection [6].

In the current study, we compared 3D-Anwar and 2D-Wilkins MV scores in assessment of the MV before percutaneous BMV and investigated the additive value of real-time 3DE in the prediction of immediate adverse outcome of percutaneous BMV. No significant difference was observed in the measured pre-procedural MVA between 2D-planimetry, PHT, and 3D-planimetry. Although such results were concordant to those of Messika-Zeitoun et al. [21, 22], but Chu et al. demonstrated that PHT had overestimated the MVA compared to continuity equation (CEQ) (*p* = 0.037), whereas no significant difference was observed between 3DE and CEQ (*p* = 0.61) [23]. Moreover, Schlosshan et al. showed that MVA by 3D-TEE measurements were significantly lower compared to it by 2D-TTE (*p* = 0.005) and PHT (*p* < 0.0001), but marginally greater than MVA by CEQ (*p* = 0.82) [24]. Also Min et al. had considered 3D-TEE for accurate MVA assessment because of overestimation of MVA by 2D-planimetry [25].

We observed a significant difference between 2DE and 3DE regarding MV score (for total score and each of its component) in cases with suboptimal post-procedural MVA or significant MR**,** while this was not observed in cases with optimal post-procedural results. Whereas 57% and 43% of them had moderate and mild total 2D-Wilkins score respectively, while 86% had severe total 3D-Anwar score and only 14% had moderate score. This could be explained by the underestimation of MV score especially the degree of calcification and subvalvular apparatus affection by 2DE, while 3DE allowed for better visualization of MV and subvalvular apparatus from the ventricular perspective, which gave a respective quantification of leaflet motion and calcification, chordate shortening and fusion, commissural union, and papillary muscle fibrosis [6]. A significant negative correlation was obtained between 3D score and the achieved post-procedural MVA, which was not obtained with 2D score. These results were comparable with those of Anwar et al. who demonstrated the superiority of 3DE over 2DE in estimation of MV score, as 10% of cases had high 2D-Wilkins score while this percent increased up to 16% by 3D-Anwar score. Moreover; they found that 3DE score components for leaflet mobility and subvalvular thickening were independent predictors of procedural success (*p* = 0.004 and 0.04 respectively). They also observed that the high mean trans-mitral gradient in 16.6% of patients with procedural success was in those with moderate or severe subvalvular score by 3DE despite being mild by 2D-wilkins score. Meanwhile, calcification was the only independent predictor for development of significant MR, with four out of 17 of those patients had severe 3DE score while having it mild by 2D-Wilkins score [6]. Nevertheless, discordant results were demonstrated by Aslanabadi et al. that 2D calcification score was useful enough for identifying patients likely to have MR development or increase after BMV [26]. Moreover, Rifaie et al. had also demonstrated that using 2DE scores that basically depended on calcification (especially commissural) and subvalvular involvement could correlate well with poor outcome in BMV patients [27].

In the current study, ROC curve analysis revealed better prediction for occurrence of suboptimal results by 3D-Anwar score (> 14) than 2D-Wilkins score (> 8). Moreover, better post-procedural MVA was obtained when excluding the six cases with suboptimal achieved MVA, those who might be excluded according to their high 3D-Anwar score.

The assessment of leaflet calcification and its distribution along leaflets’ parts play an important role in determining the outcome of BMV, as the percentage of success decreases and the incidence of complication increases with increased extent of calcification. 3DE could predict the extent and distribution of calcification in each scallop from single short axis cut plain; meanwhile, multiple cut planes are needed for detecting calcification in all scallops of both MV leaflets by 2D-Wilkins score [28]. Calcification of commissures is one of the strong predictors of outcome after BMV because it affects the degree of commissural splitting. That is why in the new 3DE score, calcification at the commissural parts of leaflet was described by a higher score than the middle leaflets calcification [29].

We observed a significant improvement in anterior and posterior commissural splitting after BMV by both 2DE and by 3DE. Although no significant difference regarding pre-procedural commissural affection was observed between 2DE and 3DE, but favorable results were obtained regarding post-procedural posterior commissural splitting when evaluated by 3DE. Several studies showed the splitting of one or both commissures along the natural planes as the mechanism of dilatation in BMV [6, 30]. Concordant with our results were those of Messika-Zeitoun et al., as 2DE underestimated the degree of commissural opening after BMV in 33% of patients compared to 3DE and agreement between methods was weak (*κ* = 0.41), explaining that by easy visualization of the degree of commissural opening by 3DE [22]. Meanwhile, Anwar et al. showed comparable results regarding achieved post-procedural commissural splitting by 2DE and 3DE [6].

In the current study, favorable post-procedural posterior commissural splitting was obtained by multi-track balloon than Inoue balloon, and no significant difference regarding post-procedural achieved MVA or MR grade between both techniques. These results were concordant to those of El-sayed et al. who compared the immediate results of percutaneous mitral commissurotomy using metallic valvotome, Inoue balloon, and double-balloon techniques. Similar higher depth score for only posterior commissural splitting was observed in double-balloon group compared to Inoue balloon group (*p* = 0.006), with comparable resulting MR grades, but unlike our results they showed better achieved MVA with double-balloon technique (*p* = 0.01) [9]. This could be explained by the ability of the double-balloon to fit and simultaneously press on both commissures, while Inoue balloon exerted its pressure mainly on the anterior one [31]. Meanwhile, Oraby and Youssef showed a better bilateral commissurotomy with multi-track balloon, with higher incidence of mild MR (*p* = 0.003), while no significant differences regarding moderate to severe MR after BMV [32].

Our surprising observation that suboptimal achieved MVA was obtained with larger balloon sizes and significant MR with smaller balloon sizes may support the claim that only mitral apparatus anatomy and score that could determine the occurrence of post-procedural adverse outcome, regardless type or size of the balloon used.

## Conclusion

From the previous data, we concluded that 3DE was an accurate tool for assessing MV before percutaneous BMV, which had a valuable role in assessing MV score especially calcification and subvalvular apparatus, and correlated well with the immediate post-procedural outcome. The 3D-Anwar score appeared as a better tool that may gave detailed morphological assessment of MV, which would help in the proper selection of the therapeutic strategy for MS and predict procedural success or immediate adverse outcome than the traditional 2D-Wilkins score.

At the start of learning, complementary use of both the 2D- and 3D-scores on large-scale before BMV will facilitate future standardization of 3DE for both quantitative and qualitative assessment of the MV.

### Limitations

The study population was initially selected for BMV depending on 2D-Wilkins score only (according to the current guidelines recommendations). Perhaps it would be better to test concomitant use of 3DE and 3D-Anwar score for the initial patient selection, which might be reflected on better procedural outcome and lesser incidence of suboptimal results than those obtained.

## Additional file

Additional file 1: Anwar et al. real-time three-dimensional echocardiographic scoring system [6].

## Data Availability

The datasets generated and/or analyzed during the current study are not publicly available as included in ongoing researches, but are available from corresponding author on reasonable request.

## Abbreviations

2D: Two-dimensional
2DE: Two-dimensional echocardiography
2D-TEE: Three-dimensional transesophageal echocardiography
2D-TTE: Two-dimensional transthoracic echocardiography
3D: Three-dimensional
3DE: Three-dimensional echocardiography
AUC: Area under curve
BMV: Balloon mitral valvuloplasty
CEQ: Continuity equation
ECG: Electrocardiogram
LA: Left atrium
MR: Mitral regurgitation
MS: Mitral stenosis
MVA: Mitral valve area
PHT: Pressure half time
PSPAP: Peak systolic pulmonary artery pressure
ROC: Receiver of characteristic
SD: Standard deviation
SPSS: Statistical package for the social sciences
TEE: Transesophageal echocardiography
TTE: Transthoracic echocardiography
