# Supplementary material for: Additive value of 3D-echo in prediction of immediate outcome after percutaneous balloon mitral valvuloplasty
Source: Egypt Heart J. 2019 Sep 18;71:19. doi: 10.1186/s43044-019-0019-x (PMC6821434; doi:10.1186/s43044-019-0019-x)
Supplement: Supplementary file 1 — Anwar et al. real-time three-dimensional echocardiographic scoring system [6]. [file 43044_2019_19_MOESM1_ESM.pdf]

Anwar et al. real-time three-dimensional echocardiographic scoring system (Anwar et al., 2010)

Leaflets scoring parameters	(a) Leaflets					
	Anterior leaflet			Posterior leaflet		
	A 1	A 2	A 3	P 1	P 2	P 3
(a) Thickness (> 4mm) (0–6) *(0 = normal, 1=thickened)*	0–1	0–1	0–1	0–1	0–1	0–1
(a) Mobility (0–6) *(0 = normal, 1=restricted)*	0–1	0–1	0–1	0–1	0–1	0–1
(b) Calcification (0–10) *(0 = no, 1–2 = calcified)*	0–2	0–1	0–2	0–2	0–1	0–2
(a) Normal = 0, Mild = 1–2, Moderate = 3–4, Severe ≥5<br>(b) Normal = 0, Mild = 1–2, Moderate = 3–5, Severe ≥6						

Subvalvular apparatus scoring parameters	(b) Subvalvular apparatus		
	Proximal third	Middle third	Distal third
Thickness (0–3) *(0) = normal (<1mm), (1) = thickened (>1mm)*	0–1	0–1	0–1
Separation (0–6) *(0) = normal (>5mm), (1) = partial (<5mm), (2) = no separation*	0–2	0–2	0–2

Total Score Grading (0-31 points)	Normal MV= 0<br>Mild MV involvement= 1-8<br>Moderate MV involvement= 9–13<br>Severe MV involvement ≥14

MV: mitral valve
